# Plasma cell disorders in HIV-infected patients: epidemiology and molecular mechanisms

**DOI:** 10.1186/2050-7771-1-8

**Published:** 2013-02-04

**Authors:** Woodrow J Coker, Ashley Jeter, Henning Schade, Yubin Kang

**Affiliations:** 1 Division of Hematology and Oncology, Department of Medicine, Medical University of South Carolina, 86 Jonathan Lucas Street, Hollings Cancer Center, Room# HO307, Charleston, SC, 29425, USA

**Keywords:** HIV, AIDS, Gammopathy, Multiple myeloma, Epidemiology, Molecular mechanism, Treatment, Outcome, Hematopoietic stem cell transplantation

## Abstract

Highly active antiretroviral therapy (HAART) has significantly improved the outcome and survival of human immunodeficiency virus (HIV)-infected patients. Subsequently, long-term morbidities including cancer have become of major public health and clinical interest for this patient population. Plasma cell disorders occur at higher incidence in HIV-infected patients; however, the molecular mechanisms driving the plasma cell disease process and the optimal management for these patients remain to be defined. This article provides an up-to-date review of the characteristics and management of HIV-infected patients with plasma cell disorders. We first present 3 cases of plasma cell disorders in HIV-infected patients, ranging from polyclonal hypergammaglobulinemia to symptomatic multiple myeloma. We then discuss the epidemiology, clinical presentation, and management of each of these plasma cell disorders, with an emphasis on the molecular events underlying the progression of plasma cell diseases from monoclonal gammopathy to symptomatic multiple myeloma. We propose a three-step hypothesis for the development of multiple myeloma. Finally, we discuss the use of high dose chemotherapy and autologous hematopoietic stem cell transplantation in the treatment of HIV-infected patients with multiple myeloma. Our review includes the care of HIV-infected patients with plasma cell disorders in the current era of HAART and novel agents available for the treatment of multiple myeloma.

## Introduction

Since the introduction of highly active antiretroviral therapy (HAART) between late 1996 and early 1997, the survival of HIV-infected patients has dramatically improved [1]. In developed countries, most HIV-infected patients live several decades after their diagnosis. Due to the effectiveness of broad-spectrum antibiotics and anti-fungal agents, fewer HIV-infected patients die from life-threatening opportunistic infections. This improvement in the long-term survival of HIV-infected patients has raised public health and clinical awareness of increased risks of cancer within this population. However, HIV-infected individuals tend to be excluded from the clinical trials used to establish current practice guidelines for cancer treatment. As a result, clinical and therapy outcome data are extremely scarce for HIV-infected patients with cancer. Hematologists and medical oncologists face with the increasingly difficult challenge of providing optimal treatment for HIV patients with cancer.

HIV-infected patients have increased risks for plasma cell disorders [2-6]. Plasma cell disorders in HIV-infected patients can manifest as polyclonal hypergammaglobulinemia, monoclonal gammopathy, or symptomatic multiple myeloma (MM). Polyclonal hypergammaglobulinemia is characterized by an increased production of several different immunoglobulins and diffusely increased proteins in the gamma region on serum protein electrophoresis (SPEP). Monoclonal gammopathy of undetermined significance (MGUS) is defined by the presence of a serum monoclonal protein (M-protein) at a level < 3 g/dl, clonal bone marrow plasma cells <10%, and the absence of end-organ damage (lytic bone lesion, anemia, hypercalcemia or renal failure) related to the proliferative process [7,8]. MM is a plasma cell malignancy and is characterized by the presence of M-protein, the infiltration of clonal plasma cells in the bone marrow (≥10%) and the evidence of end-organ damage [7,8]. Understanding the characteristics and molecular mechanisms of these plasma cell disorders in HIV-infected patients has important implications in their care. In this review article, we first present our experience in managing three cases of HIV patients with different presentations of plasma cell disorders. We then provide an up-to-date review of the epidemiology and molecular mechanisms of plasma cell disorders in HIV-infected patients. We propose a three-step hypothesis in MM development.

### Case presentation

#### Case 1

A 58-year-old African American female presented with a three-month history of fatigue, poor appetite, lack of energy, and 40 lbs weight loss. Her past medical history was significant for hysterectomy and uncontrolled diabetes. Physical examination demonstrated hepatomegaly with liver palpable 2 fingerbreadths below the right costal margin. There was no palpable lymphadenopathy. Laboratory tests showed a white blood cell count (WBC) of 2.31 × 10^9^/L, hemoglobin 9.4 g/dl, platelet count 281 × 10^9^/L; creatinine 0.8 mg/dl, serum calcium 8.9 mg/dl, total protein 8.2 g/dl, and albumin 2.51 g/dl. SPEP and immunofixation (IFE) revealed 3.22 g/dl of broad-based protein band in the gamma zone and 0.73 g/dl of monoclonal immunoglobulin G (IgG)- kappa (κ) M-protein. Bone skeletal survey showed no lytic lesions. Bone marrow aspirate and biopsy showed increased number of plasma cells (15% on biopsy) with no light chain restriction. HIV screening test was positive, confirmed with Western blot analysis. The CD4 count was 15/μl and HIV viral load 934,811 copies/ml. She was diagnosed with HIV-associated polyclonal hypergammaglobulinemia with concomitant monoclonal gammopathy. She was referred to the infectious disease clinic and was started on HAART.

#### Case 2

A 45-year-old Hispanic male was referred for evaluation of laboratory finding of hypergammaglobulinemia. During a routine lab test, the patient was found to have a total protein of 12.6 g/dl and an albumin of 2.4 g/dl. He was completely asymptomatic. His past medical history was significant for HIV infection diagnosed more than two decades ago. He participated in a clinic trial with HIV gp160 vaccination soon after the diagnosis of HIV infection, but he had not been on HAART. At the time of his initial visit in the hematology-oncology clinic, his CD4 count was 703/μl and his HIV viral load was 933 copies/ml. He had hemoglobin of 10.1 g/dl but serum calcium and serum creatinine were normal. Serum IgA level was 332 mg/dl; IgG 7,182 mg/dl; and IgM 2,221 mg/dl. SPEP and IFE exhibited an IgG- κ M-protein at 3.83 g/dl on a background of polyclonal hypergammaglobulinemia. Urine protein electrophoresis of 24 h urine (UPEP) revealed excretion of κ light chain protein at 0.23 g/24 h. Bone marrow aspiration and biopsy showed 32% κ light chain restricted- plasma cells. Karyotype and Fluorescence In Situ Hybridization (FISH) analyses of bone marrow cells were normal. His β2- microglobulin was elevated at 5.3 mg/L. Based on the presence of anemia, M-protein of 3.83 g/dl and κ light chain restricted plasmacytosis in the bone marrow, he was diagnosed with MM. He was started on HAART with Atripla® (efavirenz, emtricitabine, and tenofovir). Subsequently, he was treated with thalidomide plus dexamethasone. After one cycle, the patient developed severe neuropathy and thus the chemotherapy was changed to lenalidomide plus dexamethasone. He was also on double strength trimethoprim/sulfamethoxazole for infection prophylaxis and enoxaparin for deep vein thrombosis (DVT) prophylaxis. He achieved a very good partial response after 4 cycles of lenalidomide + dexamethasone treatment.

#### Case 3

A 53-year-old Caucasian male presented with a one-month history of chest wall and back pain, and right-sided leg weakness and pain. Past medical history was significant for HIV infection and AIDS diagnosed about 27 years ago. He had a history of cytomegalovirus (CMV) retinitis, rectal condylomata, and toxoplasmosis, and he had been taking Atripla® over the last 2 decades. His CD4 count had been >1000/μl and HIV viral load undetectable. Computed tomography (CT) and magnetic resonance imaging (MRI) studies revealed enhancing soft tissue lesions involving the posterolateral right 8th rib and the posterior left 12th rib; fracture of the left posterior 7th rib; lytic lesions involving the thoracic (T) 4, T12, and lumbar (L)1 vertebral bodies, and a large lesion involving the right sacroiliac joint. CT guided biopsy of the right sacroiliac mass revealed plasma cell neoplasm with κ light chain restriction. His WBC was 8.65 × 10^9^/L, hemoglobin 12 g/dl, platelet count 433× 10^9^/L, serum creatinine 1.0 mg/dl, albumin 2.6 g/dl, and serum calcium 10.4 mg/dl. SPEP and IFE demonstrated an IgG κ M-protein at 1.64 g/dl. Bone marrow biopsy and aspiration revealed κ light chain restricted- plasma cells at 10%. FISH analysis showed 1 extra copy of 1q and a 13q14 deletion. The patient was diagnosed with MM (Stage III by International Staging System) with extensive medullary plasmacytoma. He continued his HAART and was treated with 6 cycles of VCd regimen (bortezomib, cyclophosphamide and dexamethasone). He also received a total of 30 Gy fractioned irradiation of the spine and the iliac crests. Due to the concern of possible progression in bony lytic lesions, his chemotherapy regimen was switched to VRd (bortezomib, lenalidomide and dexamethasone). After 3 cycles of VRd, the patient achieved a very good partial response. Because of the aggressiveness of his MM, the patient then underwent high dose chemotherapy and autologous hematopoietic stem cell transplant (HSCT). He received pegfilgrastin for stem cell mobilization and successfully collected 8.3 × 10^6^ CD34^+^ cells/kg body weight with only one-day leukapheresis. He received high dose melphalan (200 mg/m^2^) conditioning and autologous HSCT. His HSCT was relatively uneventful. He had an appropriate count recovery, and restaging at 3 months post HSCT showed no evidence of M-protein in SPEP and UPEP.

## Discussion

Plasma cell disorders in HIV-infected patients range from polyclonal hypergammaglobulinemia, to monoclonal gammopathy, to malignant plasma cell neoplasms such as MM and plasma cell leukemia. Our first case represented mixed polyclonal hypergammaglobulinemia and monoclonal gammopathy commonly seen in HIV-infected patients. Our second and third cases represented malignant plasma cell neoplasms. We will discuss each of these presentations focusing on the epidemiology and mechanisms of the diseases.

### Epidemiology/incidence

#### Polyclonal hypergammaglobulinemia

Polyclonal hypergammaglobulinemia is a common finding in HIV-infected patients [3,9,10]. Konstantinopoulos, et al. performed protein electrophoreses on and measured immunoglobulin concentrations in the serum samples of 320 consecutive HIV-infected patients at the Beth Israel Deaconess Medical Center and found that 1.9% of the HIV-infected patients had polyclonal hypergammaglobulinemia [11].

#### Monoclonal gammopathy

The incidence of monoclonal gammopathy in HIV-infected patients appears higher than that in the general population, where it occurs at 3.2% among persons 50 years of age or older [12]. In HIV-infected patients, the incidence of monoclonal gammopathy ranged from 3.8% to 26% in several small retrospective studies [3,6,13,14]. Furthermore, compared to the general population, monoclonal gammopathy in HIV-infected patients occurs at a much younger age. The mean age of HIV-associated monoclonal gammopathy has been reported at 34–43 years of age [6,15]. In contrast, the mean age at diagnosis for MGUS in the general population is 70 years [12,16].

#### Multiple myeloma

There were six large population studies that demonstrated an increased incidence of MM in HIV/AIDS (acquired immune deficiency syndrome) patients [17-22]. Two of these studies were performed in the United States and covered 11 regions in the periods from 1980 to 1996 (a total of 302,834 AIDS patients) [17] and from 1996 to 2002 (a total of 375,933 AIDS patients) [19]. The standardized incidence ratios (SIRs) for MM for these two periods of time were 2.60 (95% CI: 1.92-3.44) and 2.20 (95% CI: 1.10-3.94), respectively. Grulich, et al. performed an epidemiological study of the incidence of cancers in HIV/AIDS patients in Australia nationwide from 1985 to 1999 [18]. There were a total of 13,067 HIV/AIDS patients in Australia during this period of time. The SIR for MM was 4.17 (95% CI: 1.35-9.72). The SIRs for MM in HIV/AIDS patients in Italy and Switzerland were 4.84 (95% CI: 1.00-14.14) and 5.00 (95% CI: 0.61-18.06), respectively [20,21]. Using data obtained from the Communicable Disease Surveillance Centre’s national HIV database and the Thames Cancer Registry, Newnham, et al. assessed the risk of cancers in 26,080 HIV-infected people in southeast England [22]. The SIR was significantly increased for MM (2.70 with 95% CI: 1.00 – 5.94). Grulich, et al. then performed a meta-analysis combing all six studies and found that the SIR for MM in HIV/AIDS patient was 2.71 with 95% CI of 2.13-3.44 [23]. Similarly, another meta-analysis also revealed an increased risk of MM in HIV/AIDS patients: the relative risk of MM in HIV/AIDS patients was in the range of 1.9 to 6.5 [24].

### Mechanisms/etiology

The exact mechanisms for the increased risk of plasma cell disorders in HIV patients remain poorly understood. Two main mechanisms likely contribute to the development of plasma cell disorders in this patient population: antigen stimulation and immunodeficiency.

#### Antigen stimulation

HIV viral antigens and/or other bacterial/viral antigens may act as super-antigens [3,9,10,25-27] and stimulate the proliferation of B cells and the secretion of immunoglobulin without the help of T cells. It is generally believed that chronic antigenic stimulation is the driving force for the process. Very little, however, is known about the nature of the antigen(s), and published data have been controversial. It was stipulated that viral infection or viral antigens from HIV per se, Epstein-Barr (EB) virus, Kaposi’s sarcoma virus, herpes viruses, and hepatitis B and C viruses might play an important role in driving B cell expansion.

##### HIV viruses/viral antigen

The role of HIV viruses or HIV viral antigens in the clonal expansion of B cells and the development of MM has been investigated [28,29]. Currently, there is little evidence of the HIV virus playing a direct role in the development of MM. However, B cell clonal expansion was observed in HIV-infected patients [29], and the monoclonal paraproteins in HIV-infected patients may be directed against HIV viral antigens [30-33]. This suggests that HIV viruses could play a role in the pathogenesis of plasma cell disorders. It should be noted, however, that HIV viruses can neither infect B cells or plasma cells, nor drive their malignant transformation [28].

##### Epstein-Barr virus

Voelkerding, et al. reported a case of aggressive MM in a 31-year-old HIV-infected male patient presenting with diffuse myeloma infiltration and hypercalcemia [34]. Interestingly, DNA hybridization showed the presence of EB virus genomes in myeloma tissue but not in non-tumor tissue [34]. Additionally, Kumar, et al. reported 3 cases of MM in young HIV-positive males, with two of these cases demonstrating a clonal population that was positive using a probe to the EB virus terminal repeat region [35]. These small studies suggest a potential association between MM and EB virus. However, additional studies are needed to confirm this connection between the EB virus and MM pathogenesis.

##### Human herpes virus 8 (HHV8)

HHV8 is a gamma herpes virus and causes Kaposi’s sarcoma (KS) in HIV/AIDS patients. Cannon, et al. examined the correlation between MM and KS [36]. The authors obtained cancer incidence and survival data from the US Surveillance, Epidemiology, and End Results (SEER) program between 1973 and 1995. They found that the vast majority of MM cases were most likely not associated with KS or HHV8. Using polymerase chain reaction (PCR) analysis, HHV8 DNA was found positive in KS biopsies, but not in MM bone marrow biopsies [37,38]. No antibodies against HHV8 were found in sera from MM patients [39-41]. None of these studies support a role for the HHV8 in the genesis of MM. While the data strongly support the conclusion that HHV8 is not connected to the MM pathogenesis, an alternative study conducted by Chauhan et al., in which the scientists used nested PCR to detect HHV8 DNA sequences in bone marrow stromal cells from 26 patients with MM, and found that the majority of these MM patients (92%) had detectable KS DNA in the long-term bone marrow stromal cells [42]. The implications of Chauhan’s findings are unclear.

#### Immunodeficiency

HIV viruses can cause T cell dysfunction, and dysfunctional T cells may induce the activation of B cells without the need for antigen stimulation [43]. Additionally, HIV infection depletes T cells resulting in profound immunodeficiency. Grulich, et al. recently performed a meta-analysis to investigate the role of immunodeficiency in MM development. The authors compared the incidence of cancers including MM in HIV/AIDS patients and in kidney transplant patients receiving immunosuppression [23]. The authors reasoned that, while both of these populations were immunosuppressed, kidney transplant patients would differ substantially from HIV/AIDS patients in lifestyle-related cancer risk factors. If cancer incidence patterns were similar between the two populations, then one could conclude that immune deficiency was primarily responsible for the increased risks of cancers in HIV/AIDS patients. Indeed, the authors found that the SIR for MM in HIV/AIDS patients was 2.71 (95% CI: 2.13-3.44), which was quite similar to that in renal transplant recipient (SIR: 3.12 and 95% CI: 2/13-4.57). These data suggest that T cell depletion and immune deficiency may play a key role in the increased incidence of MM in HIV/AIDS patients.

### Molecular mechanisms of myelomagenesis

Recent advancements in molecular biology and cancer genomics technology have tremendously improved our understanding of the molecular pathways involved in the pathogenesis of MM. The development of normal plasma cells involves 2 main organs: the bone marrow and the lymph node [44]. In the bone marrow, the common lymphocyte progenitor cells differentiate into pro-B cells that undergo immunoglobulin heavy chain (IgH) gene rearrangement and differentiate into pre-B cells. Pre-B cells express cytoplasmic mu chains but no surface immunoglobulin due to minimal light chain gene rearrangement. Once light chains rearrange, surface IgM can be expressed on immature B cells. These cells migrate out of the bone marrow and into the lymph node and other peripheral lymphoid tissues. In the lymph node, the IgM+ B cells encounter antigen (and receive T-cell help), and then can undergo somatic hypermutation. After activation, B cells then become either memory B cells that when encountering the same antigen mount a rapid and robust immune response or plasma cells that produce antibodies to bind and neutralize the antigen. Recent studies suggest that Interferon Regulatory Factor 4 (IRF4, also called Mum1) plays an instrumental role in the differentiation of B cells into plasma cells [45]. Once IRF4 is expressed and activated, the B cells initiate plasmacytic differentiation. Additionally, IRF4 up-regulates Blimp1 and down-regulates Bcl-6. The decrease in Bcl-6 and increase in Blimp-1 further drive B cell differentiation into plasma cells [46].

**Figure 1:**
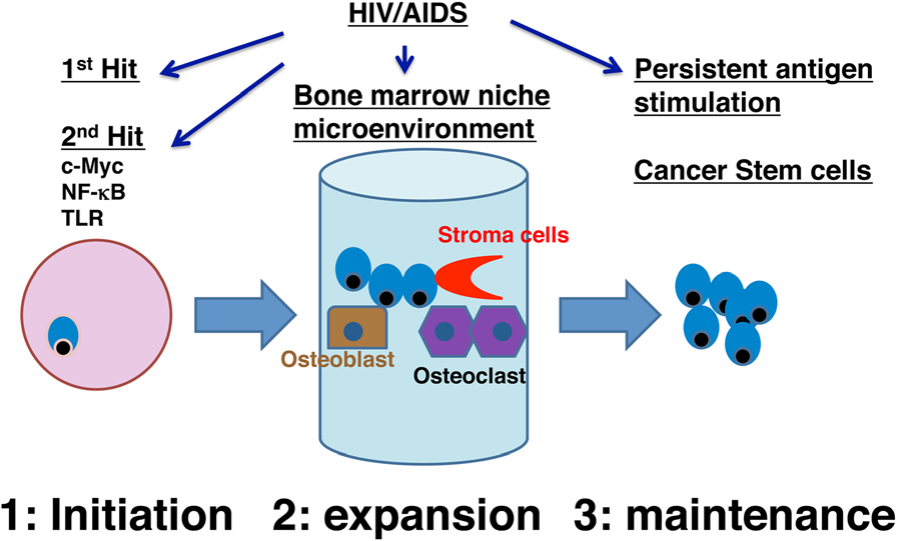
**A 3-step working hypothesis for the development of multiple myeloma.** HIV infection contributes to the development of MM likely through several different mechanisms: 1) HIV infection increases the risk for somatic hypermutation; 2) HIV infection activates cell survival pathways; 3) HIV infection alters bone marrow niche microenvironement; and 4) HIV infection causes persistent antigen stimulation.

We hypothesized that the development of MM is a 3-step process that involves many signaling pathways (Figure 1). The first step in the MM pathogenesis is the initiation step, in which normal plasma cells become malignant myeloma cells. The second step - the expansion step - occurs when malignant myeloma cells interact with bone marrow niche microenvironment(s), resulting in expansion, proliferation and dissemination of myeloma cells. The third step is the maintenance step where a rare population of myeloma stem cells persists despite currently available treatment. These cells repopulate, leading to a relapse of the disease. In the following paragraphs, we will review some of the important molecular mechanisms and pathways that occur during each of these steps. HIV infection likely has multifaceted impacts on myelomagenesis (Figure 1).

#### Step 1: initiation step

How plasma cells become malignant myeloma cells is not yet fully understood. However, numerous studies support a two-hit theory in MM development. The first hit occurs during the process of somatic hypermutation of B cells in the lymph node. It is postulated that the first hit results in the transformation from normal plasma cells to light chain restricted- clonal plasma cells that cause MGUS. The first hit leads to 2 types of molecular changes: hyperdiploid change with multiple trisomies, or non-hyperdiploid change. The non-hyperdiploid change commonly involves the translocation of IgH chain gene at 14q32 with one of the five translocation partners: cyclin D1 at 11q13; cyclin D3 at 6p21; transcription factor c-maf at 16q23; fibroblast growth factor receptor (FGFR) 3 and histone methyltransferase MMSET at 4p16; and transcription factor mafB at 20q11 [47,48]. Deletion of chromosome 13 is a common non-hyperdiploid change.

After the first hit, the clonal B cells/plasma cells egress out of the lymph node and circulate in the blood stream and seed into the bone marrow. In order for the clonal B cells/plasma cells to become malignant myeloma cells, there must be a second hit that promotes cell growth and proliferation. This second hit involves activation of the myelocytomatosis virus oncogene cellular homolog (c-MYC) pathway, FGFR 3, kirsten rat sarcoma viral antigen (KRAS) and neuroblastoma (N) RAS pathway, and nuclear factor (NF)-κB pathway; loss of function in histone demethylase; activation of toll-like receptor (TLR) signaling; and stimulation from bone marrow microenvironment. In this review article, we focus on c-MYC and NF-κB pathways, TLR signaling, and the bone marrow microenvironment.

The c-Myc oncogene plays a key role in cell proliferation, growth, differentiation, and apoptosis [49,50]. c-Myc is dysregulated or over-expressed in MM and plays a critical role in the progression from MGUS to MM [51,52]. The progression of MGUS to myeloma is associated with several-fold increase in MYC RNA expression [53]. Overexpression of MYC leads to the development of myeloma phenotypes in the Vk*MYC mouse model [54]. Furthermore, targeting MYC using short hairpin RNA or a selective small molecule inhibitor of MYC-Max heterodimerization (10058-F4) induced myeloma cell death [55]. These data suggest that myeloma cells are addicted to c-MYC activity, and that c-MYC is indispensable in myeloma development [53].

NF-κB is an important transcription factor that regulates cell proliferation, angiogenesis, metastasis, inflammation, and apoptosis [56]. Ni, et al. examined the NF-κB activity in 13 primary myeloma patient samples and in four myeloma cell lines. NF-κB was constitutively active in all MM patient samples and in all four myeloma cell lines [57]. Inhibition of NF-κB induced apoptosis in both primary myeloma cells and in myeloma cell lines [57]. NF-κB has two signaling pathways: the classical NF-κB (i.e., RELA/p50 heterodimer) signaling pathway and the alternative NF-κB (heterodimers of RELB/p50 and RELB/p52) pathway [58]. Both pathways can be activated by CD40 and B-cell activating factor. The classical pathway activates via Iκβα and is involved in innate immunity, inflammation and cell survival. The alternative pathway acts through the IκB kinase α (IKKα) and is involved in lymphoid carcinogenesis, humoral immunity and B cell maturation. Disruption of NF-κB pathways sensitizes MM cells for apoptosis [59,60].

TLRs recognize pathogen-associated molecular patterns and are important in innate immunity. TLRs are expressed and activated in MM and have diverse but contradictory effects on myeloma cells [61,62]. TLR activation in MM has important consequences. 1) Myeloma cell growth: TLR activation induces myeloma cell proliferation, survival and resistance to drug-induced apoptosis through the activation of NF-κB and mitogen-activated protein kinase (MAPK) pathways [63]. 2) Immune evasion. TLR activation can up-regulate the expression of co-stimulatory molecules and B7 family members through the Myeloid differentiation primary response gene 88 (MyD88)/TNF receptor associated factor (TRAF)-6, resulting in tumor evasion from the immune response [64]. 3). Production of pro-inflammatory cytokines.TLR activation induces human mononuclear cells and primary myeloma cells to secrete pro-inflammatory cytokines including IL-3, IL-6, vascular endothelial growth factor, basic fibroblast growth factor, hepatocyte growth factor, macrophage inflammatory protein (MIP)-1α, and the receptor activator of NF-κB ligand (RANKL) [65]. 4). Up-regulation of adaptor molecules. TLR activation induces the expression of most adaptor molecules including MyD88, TIR-domain-containing adapter-inducing interferon-β (TRIF) and TRAF-6 [66].

Recently, the whole genome or exome of 38 myeloma patients were sequenced [67]. The frequency of tumor-specific point mutations was 2.9 per million bases, corresponding to approximately 7,450 point mutations per sample across the genome. Extensive somatic mutations were found in MM patients, including mutations of genes involved in protein translation (seen in nearly half of the patients), histone methylation, and blood coagulation. In addition, mutations in 11 members of the NF-κB pathway were found, further supporting the important role of NF-κB in myeloma pathogenesis [67]. More recently, Keats, et al. performed genomic analysis on myeloma cell samples collected at different points throughout the course of disease of 28 different patients. They found that the genome of patients with cytogenetically high-risk MM tended to show significant changes over time [68]. Similarly, Egan, et al. conducted a longitudinal whole-genome sequencing of four tumor samples collected from a single myeloma patient at various stages of the disease and found that the genomic sequences of the tumor varied as the disease progressed [69]. Walker, et al. used whole exome sequencing to define the mutational landscape between t(4;14) and t(11;14) myeloma patients. The authors found that there is a distinct mutational landscape in these two groups of patients, and that each group follows a distinct pathway [70]. Subsequently, Bahlis proposed a Darwinian-like somatic evolution in myeloma tumor progression [71].

#### Step 2: expansion step

During Step two of myeloma pathogenesis, c-Myc, NF-κB, TLR, KRAS, NRAS, and many other mechanisms that play a key role in the myeloma initiation step continue to promote myeloma cell proliferation and expansion. The interaction between myeloma cells and the bone marrow microenvironment, which include bone marrow stromal cells, osteoblasts and osteoclasts, leads to the expansion of myeloma cells and the manifestations of end-organ damages [72-75].

After the first and/or the second hit, myeloma cells up-regulate the expression of surface adhesion molecules including CXCR4, E-cadherin, Integrin α4β1 (also called very late antigen-4, VLA-4), leukocyte function-associated antigen-1 (LFA-1), vascular cell adhesion molecule 1 (VCAM-1), intracellular adhesion molecule-1 (ICAM-1), etc. These changes in surface adhesion molecules allow the myeloma cells to home to the bone marrow microenvironment [76]. Once these myeloma cells enter the bone marrow, they adhere to the bone marrow stromal cells through the interaction of CXCR4/stromal derived factor (SDF)-1, VLA-4/VCAM-1, LFA-1/ICAM-1, or others. The adhesion of myeloma cells to the stromal cells prompts the bone marrow stromal cells to secrete osteoclast-activating factors (OAFs) such as IL-1β, IL-6, and TNF-β, which further induce stromal cells and osteoblasts to secrete RANKL. RANKL activates osteoclast progenitors, and the increased osteoclastic activity leads to release of several cytokines – such as transforming growth factor-β (TGF-β), IL-6, bFGF, and insulin-like growth factors – from the bone matrix. These cytokines directly or indirectly stimulate myeloma cell growth and cause the MM cells to release parathyroid-hormone-related protein, which further induces the secretion of RANKL. The end result is a vicious circle, with MM cells stimulating bone resorption, and bone resorption leading to increased tumor growth [77].

Myeloma cells also play a critical role in their own expansion. Myeloma cells secrete Dickkopf-related protein 1 (DKK-1), IL-3, HGF and TGF-β to inhibit osteoblasts, and they secrete RANKL, TNF, lymphotoxins, SDF-1 and MIP-1α to activate osteoclasts.This inhibition of osteoblasts and activation of osteoclasts results in increased levels of bone-derived tumor growth factors that further promote the expansion and proliferation of myeloma cells [78]. The direct contact between myeloma cells and osteoclasts promotes myeloma cell growth [79].

Myeloma cells constantly egress out of bone marrow, circulate in the blood stream, and reseed back to the bone marrow, leading to the spread and expansion of myeloma cells. A recent study by Azab, et al. [80] suggested that hypoxia and epithelial- mesenchymal transition (EMT) play an important role in this process. Hypoxia is caused by the growth of myeloma cells in the bone marrow, and activates EMT-related machinery in myeloma cells. The EMT transition decreases the expression of E-cadherin and the adhesion of myeloma cells to the bone marrow, while leading to the egression of MM cells into the blood stream. On the other hand, hypoxia increases the expression of CXCR4, enhances the homing of circulating myeloma cells to new bone marrow niches, and results in MM dissemination and expansion.

#### Step 3: maintenance step

MM is still considered an incurable disease. Eventually, almost all patients tend to relapse and develop resistance to currently available treatment. The Step three in MM pathogenesis is to maintain the presence of myeloma cell population likely through 3 mechanisms: 1) Persistent presence of antigen stimulation to continuously drive the disease process; 2) Protection of myeloma cells in the marrow niche microenvironment from chemo treatment [81]; and 3) Presence of clonogenic myeloma cancer stem cells.

Pilarski, et al. first documented the presence of clonogenic myeloma cancer stem cells in human MM samples using serial transplant models in NOD mice [82]. Matsui, et al. found that the frequency of clonogenic myeloma stem cells is in the range of ~ 1 in 1,000 to 1 in 100,000 cells (similar to hematopoietic stem cells). These clonogenic myeloma cancer stem cells show normal memory B cell phenotypes (i.e., CD138^neg^CD20^+^CD27^+^). Not only are they resistant to dexamethasone, lenalidomide, and bortezomib and display Hoechst 33342 side-population stem cell properties, but they also have significantly higher levels of aldehyde dehydrogenase (ALDH) activity and are quiescent (in G_0_-G_1_ resting stage). Furthermore, these cancer stem cells have the ability to self-renew and differentiate [83,84]. It has been further demonstrated that the hedgehog signaling pathway is critical in maintaining the myeloma cancer stem cell population [85].

### Clinical characteristics

HIV patients with polyclonal hypergammaglobulinemia commonly present with elevated levels of immunoglobulins and diffusely increased proteins in the gamma regimen of SPEP. A bone marrow biopsy is usually not required, but if performed, these patients show plasmacytosis without light chain restriction. Patients with monoclonal gammopathy show a narrow, but distinct protein spike on SPEP and/or UPEP and may also have a background polyclonal hypergammaglobulinemia. Serum free light chain may also be elevated in these patients. Most importantly, MGUS patients show no evidence of end organ damage.

Briault, et al. utilized immunoelectrophoretic analysis and immunoblotting techniques to characterize the monoclonal immunoglobulin in HIV-infected patients [14]. Interestingly, the predominant light chain in HIV-related monoclonal immunoglobulin was lambda (λ) type (the κ:λ ratio in the whole series: 0.6). There was no monoclonal IgA immunoglobulin in this series of patients. The subclass distribution of monoclonal IgG in HIV-associated monoclonal gammopathy was different from that observed in MM patients in the general population. HIV-infected patients displayed a much higher frequency of IgG3 and IgG4 M-proteins, and a much lower frequency of IgG1 M-proteins. In these patients, the distribution of monoclonal IgG1, IgG2, IgG3 and IgG4 were 42.5%, 19%, 23.5%, and 15%, respectively. In MM patients in general population, the distribution for IgG1, IgG2, IgG3 and IgG4 were 76.1%, 14.2%, 3.4%, and 6.3%, respectively.

In the general population, MGUS progresses to symptomatic plasma cell neoplasms or lymphoproliferative disorders at a risk of 0.6 to 3.4 percent/year [86]. The risk of progression can be stratified using 3 adverse factors; i.e., the level of serum M-protein, the type of serum M-protein, and the ratio of serum free light chains [87,88]. It is unclear if the monoclonal gammopathy seen in HIV-infected patients may also represent a premalignant clonal plasma cell disorder that can progress to malignant plasma cell neoplasm. It remains to be determined if the risk-stratification model used in the general population for MGUS transformation is applicable in HIV-infected patients. Lefrere, et al. followed eleven HIV patients with monoclonal gammopathy over a 6-year period of time and found that none of these patients developed an overt plasma cell malignancy or non-Hodgkin’s lymphoma [13]. Similarly, a smaller study with only seven HIV patients with monoclonal gammopathy also did not show any progression to NHL or plasma cell malignancy over a 2–4 year period [33]. However, due to the small patient population and short follow-up of these studies, more data are needed before we can draw any definitive conclusions.

MM in HIV/AIDS patients has several unique characteristics. 1) In the general population, MM is a disease for older adults: the median age at diagnosis is 66 years and only 2 percent of patients are younger than 40 years [89,90]. For HIV-infected patients, on the other hand, MM occurs at a much younger age: the mean age of HIV patients with MM in a series of 35 patients was 42 years [90]. 2) MM in HIV-infected patients shows an atypical clinical evolution; it tends to present as solitary bone plasmacytoma or extramedullary plasmacytoma [91]. These patients also tend to have low level of M-protein despite the aggressiveness of the disease. Some patients may present as plasma cell leukemia. 3) The progression of MM in HIV-infected patients is very rapid and the overall survival is short. 4) MM in HIV-infected patients shows atypical histopathological findings, and some patients may present with anaplastic features. These anaplastic myeloma cells are negative for the common leukocyte antigen, lysozyme, and the cytoplasmic immunoglobulins. Anaplastic MM can present as the first manifestation of AIDS [92,93]. 5) MM in HIV-infected patients tends to occur in anal sex patients. 6) Elevated serum LDH level correlate with a poorer outcome in HIV/AIDS patients with MM.

The interval between HIV infection and diagnosis of MM remains to be determined. In both our patients, MM was diagnosed more than 2 decades after HIV infection (Case 3 developed AIDS, and case 2 did not have AIDS). It is important to note that there have also been reports of MM as the first presentation of HIV/AIDS [92]. Therefore, it appears that MM occurs at various stages of HIV infection: some patients develop MM during the early stage of HIV infection, while the others will develop MM several decades later.

### Management

#### General principles

The management of plasma cell disorders in HIV-infected patients is quite similar to that in the general population. For HIV-infected patients with polyclonal hypergammaglobulinemia and monoclonal gammopathy, the current standard of care is close follow-up and HAART if indicated. For HIV-infected patients with symptomatic MM, the appropriate management is to initiate chemotherapy and continue on HAART.

The hallmark of MM is the existence of end-organ damage (i.e., hypercalcemia, renal insufficiency, anemia, or bone lytic lesions), which distinguishes symptomatic MM from smoldering MM and MGUS [94]. Because HIV infection or HAART can also cause anemia or renal insufficiency, we find it important to ascertain the causes for anemia and renal insufficiency in these patients. In our practice, we usually consider punched out lytic lesion and hypercalcemia relatively unique to MM. For anemia and renal insufficiency, we usually pursue additional workup to determine the underlying cause(s) before we initiate chemotherapy.

#### HAART

HAART is important in the management of plasma cell disorders in HIV-infected patients. There are limited data that suggest that a good response to HAART may lead to M-protein reduction in some HIV-infected patients with monoclonal gammopathy [95]. For instance, nine out of 25 patients had a decrease in serum M-protein level while receiving HAART [15]. Polyclonal hypergammaglobulinemia may resolve in some patients after HAART [96]. However, it remains to be determined if HAART treatment will delay the progression from monoclonal gammopathy to an overt plasma cell malignancy [97].

HAART was introduced to developed countries between late 1996 and early 1997. Thus, the effects of HAART in the incidence of MM in HIV/AIDS patients could be potentially determined by comparing the SIRs before 1996 and after 1996. Frisch, et al. examined the SIRs of MM in 302,834 AIDS patients during the period from 1980 to 1996. The SIRs were calculated between 60 months before the development of AIDS and 27 months after AIDS. The SIR for MM from 1980 to 1996 was 2.60 (95% CI: 1.92 -3.44) [17]. In a follow-up study covering the same regions of population, Engels, et al. examined the SIRs of MM from 1996 to 2002. There were a total of 375,933 AIDS patients and the SIRs were calculated between 4 to 27 months after AIDS. The SIR for MM from 1996 to 2002 was 2.20 (95% CI: 1.00-3.94) [19]. These two studies comparing the SIRs before and after 1996 suggest a possible positive impact of HAART on the incidence of MM in HIV/AIDS patients. However, this impact appears moderate and suggests that HAART per se does not eliminate the occurrence of MM in HIV-infected patients.

In our own practice, all HIV-infected patients with MM who have not been on HAART are referred to the infectious disease service as soon as possible to initiate HAART prior to chemotherapy. HAART is initiated regardless of the patient’s history of opportunistic infection or CD4 count. For instance, our patient #2 had a CD4 count of 703/μl with no history of opportunistic infection; he was started on HAART prior to initiation of chemotherapy because of the risks for further immunosuppression and bone marrow suppression. The exact effects of HAART on myeloma outcome remain to be determined. There have been reports that HAART alone can lead to the complete remission of smoldering multiple myeloma [98]. However, these reports have been unconfirmed. It is also unclear how long the patients need to be on HAART before chemotherapy is initiated. Our patient (case#2) started chemotherapy almost immediately after the initiation of HAART. For HIV/AIDS patients with MM who have been on HAART, we continue HAART throughout the course of chemotherapy.

It was recently reported that protease inhibitors – such as ritonavir, saquinavir, and nelfinavir (but not indinavir) - induced growth cell arrest and apoptosis in several human myeloma cell lines as well as in primary myeloma cells. These protease inhibitors down-regulate the antiapoptotic protein Mcl-1, block interleukin-6-stimulated phosphorylation of STAT3, and inhibit production of vascular endothelial growth factor [99]. Nelfinavir has synergistic effects with bortezomib on the proteotoxic death of myeloma cells [100]. These data support our belief that all the HIV-infected patients with MM should be on HAART during chemotherapy.

#### Chemotherapy for MM

Clinical data on MM in HIV/AIDS patients are sparse and do not allow for detailed evaluation of therapy or the outcome of treatment on disease-free survival or overall survival. Furthermore, most HIV-positive patients with MM are excluded from clinical trials. There is currently a lack of well-supported practice guidelines for managing this specific population of patients. The treatment for HIV-infected patients with MM is largely extrapolated from data obtained from the HIV-negative population of patients.

Chemotherapy regimens for MM in HIV-infected patients are the same as those for HIV-negative MM patients, and usually consist of 2 to 3 drug combinations of thalidomide, lenalidomide, bortezomib, and dexamethasone [101]. The regimens we used for HIV-infected MM are: Td (thalidomide + dexamethasone), Rd (lenalidomide + dexamethasone), Vd (bortezomib + dexamethasone), VRd (bortezomib + lenalidomide + dexamethasone), and VCd (bortezomib + cyclophosphamide + dexamethasone). The dosing regimens and adjunctive treatment follow the NCCN recommendations and guidelines. For instance, DVT prophylaxis is used along with regimens consisting of thalidomide or lenalidomide. Herpes zoster prophylaxis with acyclovir is used in regimens with bortezomib.

Aboulafia, et al. reported a combination of thalidomide, dexamethasone, and clarithromycin for the treatment of an HIV-associated MM patient. The regimen led to a rapid and dramatic antitumor effect with modest regimen-related toxicities. The patient retained a normal CD4 count and a non-detectable HIV viral load. Due to the purported immunologic benefit of thalidomide, the author proposed further evaluation of thalidomide-based regimens in the treatment of HIV-positive myeloma patients [102]. Elira Dokekias, et al. reported using VMCP and VAMCP regimens for the treatment of HIV-positive myeloma patients. The regimens were well tolerated without major complications [103]. In our center, we have adopted a uniform treatment regimen (i.e., VCd: bortezomib + cyclophosphamide + dexamethasone) for all newly diagnosed MM patients and we have achieved ~95% overall response rate [104].

#### Autologous hematopoietic stem cell transplantation (HSCT)

The role of autologous HSCT in HIV-positive MM patients is unclear. There have been reports of high dose chemotherapy and autologous HSCT for MM in HIV-infected patients. For instance, Kentos, et al. reported one case of autologous CD34-positive blood HSCT for an AIDS patient with MM [105]. The patient had successful stem cell mobilization and collection and tolerated the transplant uneventfully with appropriate stem cell engraftment. Unfortunately, the patient died one year post transplant due to relapse of his MM [105]. This case report demonstrates the feasibility of high dose chemotherapy and autologous HSCT in HIV-infected myeloma patients. Gorschluter, et al. performed a retrospective study measuring CD4+ lymphocyte counts after autologous HSCT and found that opportunistic infection was rare post HSCT in HIV-infected myeloma patient population [106]. Our case #3 proceeded with high dose melphalan conditioning and autologous HSCT after achieving a very good partial response from induction chemotherapy. He achieved appropriate engraftment of all cell lineages after HSCT and showed no evidence of M-protein on SPEP and UPEP three months post HSCT. These data suggest that given the aggressive nature of MM in HIV-infected patients, high dose chemotherapy and autologous HSCT should be considered in young patients with good performance status.

## Conclusion

Plasma cell disorders occur at an increased frequency in HIV-infected patients and range from polyclonal hypergammaglobulinemia to aggressive MM. The development of MM involves diverse molecular mechanisms and pathways, and understanding these pathways has important implications in the treatment of MM in general. The widespread use of novel therapeutic agents and the incorporation of high dose chemotreatment followed by autologous HSCT have improved the outcome of patients with MM. However, the effects of these treatment options in HIV-infected MM patients remain to be determined.

### Consent

Consents were obtained from the patients for publishing these data.

## Competing interests

The authors declare no competing financial interests.

## Authors’ contributions

Drs. WJC, AJ, and YK participated in the care of the patients. Drs. WJC, HS, and YK wrote the manuscript. All authors read and approved the final manuscript.

## Authors’ information

Drs. Coker and Jeter are hematology-oncology fellows at the Medical University of South Carolina. Dr. Schade is a second year resident at the Department of Medicine, Medical University of South Carolina. Dr. Kang is an assistant professor and attending physician at the Division of Hematology-Oncology, Medical University of South Carolina.
